# Understanding regional activation of thoraco-lumbar muscles in chronic low back pain and its relationship to clinically relevant domains

**DOI:** 10.1186/s12891-021-04287-5

**Published:** 2021-05-11

**Authors:** Francesca Serafino, Marco Trucco, Adele Occhionero, Giacinto Luigi Cerone, Alessandro Chiarotto, Taian Vieira, Alessio Gallina

**Affiliations:** 1 Presidio Sanitario San Camillo, Torino, Italy; 2 Montecatone Rehabilitation Institute, Imola, BO Italy; 3 Degree course of Physiotherapy, Universitá degli Studi di Torino, Torino, Italy; 4 Laboratory for the Engineering of the Neuromuscular System, Politecnico of Torino, Torino, Italy; 5 PoliTo BIO Med Lab, Politecnico di Torino, Torino, Italy; 6 Department of General Practice, Erasmus MC, University Medical Center, Rotterdam, the Netherlands; 7 Department of Health Sciences, Faculty of Science, Vrije Universiteit Amsterdam, Amsterdam Movement Sciences, Amsterdam, the Netherlands; 8 Centre of Precision Rehabilitation for Spinal Pain, School of Sport, Exercise and Rehabilitation Sciences, University of Birmingham, Birmingham, United Kingdom

**Keywords:** Low back pain, neuromuscular adaptation, electromyography, clinical outcomes

## Abstract

**Background:**

Altered regional activation of the lumbar extensors has been previously observed in individuals with low back pain (LBP) performing high-effort and fatiguing tasks. It is currently unknown whether similar alterations can be observed during low-effort functional tasks. Similarly, previous studies did not investigate whether side differences in regional activation are present in individuals with LBP. Finally, there is limited evidence of whether the extent of the alteration of regional activation is associated with clinical factors. Therefore, the aim of this study was to investigate whether individuals with LBP exhibit asymmetric regional activation of the thoraco-lumbar extensor muscles during functional tasks, and if the extent of neuromuscular control alteration is associated with clinical and psychosocial outcome domains.

**Methods:**

21 participants with and 21 without LBP performed five functional tasks (gait, sit-to-stand, forward trunk flexion, shoulder flexion and anterior pelvic tilt). The spatial distribution of activation of the thoraco-lumbar extensor muscles was assessed bilaterally using high-density electromyography. For each side, the distribution of electromyographic (EMG) amplitude was characterized in terms of intensity, location and size. Indices of asymmetry were calculated from these features and comparisons between groups and tasks were performed using ANOVA. The features that significantly differed between groups were correlated with self-reported measures of pain intensity and other outcome domains.

**Results:**

Indices of asymmetry did not differ between participants with and without LBP (*p* > 0.11). The cranio-caudal location of the activation differed between tasks (*p* < 0.05), but not between groups (*p* = 0.64). Participants with LBP showed reduced EMG amplitude during anterior pelvic tilt and loading response phase during gait (both *p* < 0.05). Pearson correlation revealed that greater pain intensity was associated with lower EMG amplitude for both tasks (*R*<-0.5, *p* < 0.05).

**Conclusions:**

Despite clear differences between tasks, individuals with and without LBP exhibited similar distributions of EMG amplitude during low-effort functional activities, both within and between sides. However, individuals with LBP demonstrated lower activation of the thoraco-lumbar muscles during gait and anterior pelvic tilt, especially those reporting higher pain intensity. These results have implications in the development or refinement of assessment and intervention strategies focusing on motor control in patients with chronic LBP.

## Background

Low back pain (LBP) is the leading cause of disability worldwide [1]. This musculoskeletal disorder is also associated with high socioeconomic costs (especially chronic LBP) and long-term consequences, including decreased ability to perform activities of daily living [2, 3]. The majority of people with chronic LBP fall into the category of non-specific LBP, with wide variation in presentation and underlying pain-processing mechanisms. These pain-processing mechanisms are influenced by multiple contributors such as physical factors, psycho-social factors and their interactions [4].

Recent revisions of the literature have highlighted the importance of the role of lumbar muscles in the onset and maintenance of LBP [5, 6]. A contemporary pain-adaptation theory [7] predicts that pain chronicity and recurrence are long-term consequences of the redistribution of muscle activity that occurs to protect painful/sensitized tissues. In the case of LBP, as the lumbar erector spinae and the multifidus are activated in almost all tasks that involve spinal movements [8, 9], altered or asymmetrical activation of these muscles may change the load distribution within the lumbar spine, and hence be a primary reason for sustained pain and disability in individuals with LBP. This is supported by the fact that exercises aimed at modifying the activation patterns of the muscles acting on the spine result in clinical improvements in individuals with LBP [6, 10], and that poor motor control of the multifidus at baseline predicts response to motor control training in individuals with LBP [11]. Further studies are needed to clearly identify how LBP may affect the activation of the lumbar extensors; specifically, as pain can induce subtle redistribution of activity between regions within a muscle group [7], it is necessary to characterize the activation of regions within the lumbar extensors in people with and without LBP.

Regional activation within the low back extensors in individuals with LBP has typically been inferred from the uneven spatial distribution of the amplitude of high-density surface electromyograms (HDsEMG) [12]. Regional variations in the amplitude of EMGs collected along the lumbar spine were observed in people with LBP during repetitive lifting of a weight [13], sustained isometric contraction [14, 15] and rowing [16], but not during sitting [17]. This suggests that regional changes in activity observed during high-effort, fatiguing tasks may not be evident during low intensity tasks, such as daily-living activities. In addition, while several studies only assessed the lumbar extensor muscles unilaterally [13, 15], a recent study investigated thoraco-lumbar regional activation of within both sides [18]; however, the authors did not assess the symmetry of regional activation [19], which could be another strategy to redistribute muscle activation in pain. Determining whether regional activation of the thoraco-lumbar extensors during low-effort functional tasks activities is altered in people with LBP, and whether these alterations are symmetrical, is important to define potential targets for rehabilitation and therapeutic exercise.

Clinical and psychosocial factors are assumed to influence motor adaptation in people with LBP, their influence on the regional activation of the thoraco-lumbar extensors is unknown. Recent evidence demonstrates that biomechanical adaptations are associated with psycho-social factors [20, 21]. For instance, biomechanical responses to experimentally-induced LBP are related to pain catastrophizing behavior [20], and trunk stiffness in response to a forward perturbation correlates with kinesiophobia in people with LBP [21]. Furthermore, clinical features such as pain severity and location were also shown to be associated with the reorganization of the motor cortex in individuals with LBP [22], which is a determinant of neuromuscular activation patterns. This data suggests a relationship between neuromuscular adaptation and clinical features; however, to our knowledge only one study [14] investigated the association between regional activation of the lumbar extensors and core clinical features like pain intensity and physical functioning. A detailed investigation of the relationships between regional activation of thoraco-lumbar muscles during functional tasks and clinically relevant domains is needed to understand whether psychosocial and clinical factors are associated with the presence of altered regional activation.

In this study, we investigated whether individuals with LBP show asymmetrical regional activation, defined as side-differences in the amplitude distribution of surface EMG signals collected from the thoraco-lumbar extensors while performing functional tasks, and whether the extent of this adaptation depends on psychosocial and clinical factors. More specifically, the aims were: 1) to compare the amplitude distribution of surface EMGs collected bilaterally from the thoraco-lumbar region in patients with and without chronic LBP during different functional tasks; 2) to explore the relationship between regional changes in EMG amplitude and clinically relevant outcome domains (i.e. pain intensity, physical functioning, pain catastrophizing and self-efficacy) in patients with chronic LBP.

## Methods

This study was an observational cross-sectional study, conducted at Presidio Sanitario San Camillo of Torino and at the Laboratory of Engineering of Neuromuscular System (LISiN), Politecnico di Torino. The data collection was performed between March and October 2019.

### Participants

Twenty-one participants with chronic non-specific LBP and twenty-one asymptomatic controls were recruited. Participants with LBP were recruited through referral from physiotherapy practices and general advertising on the website of the Italian Physiotherapy Association (AIFI) Piemonte e Valle d’Aosta. Pain-free control participants were recruited from students, health workers of Presidio Sanitario San Camillo and from the community via social media advertisement and word of mouth. The study conformed to the ethical standards of the updated Declaration of Helsinki and was approved by the Ethical Committee of the “A.O.U. Città della Salute e della Scienza di Torino - A.O. Ordine Mauriziano - A.S.L. Città di Torino” (protocol n. 27560). Participants gave written informed consent prior to data collection.

To be enrolled in the study, participants had to be 18 to 65 years old. Asymptomatic controls were included if they had no lifetime history of LBP. Chronic non-specific LBP was defined as self-reported LBP of no specific origin persisting daily for at least 3 months or for at least half of the days in the last 6 months [23]. Participants with a specific cause for their LBP (e.g. herniated disk, spinal stenosis, cauda equina syndrome, infection, fracture, tumor) or symptoms radiating below the knee were excluded. Participants were excluded from both groups if they were or had recently been pregnant, or if they had any major neurological or respiratory disorders, previous spinal surgery, neurological signs (e.g. weakness, paresthesia), lower-limb pain or injury that limited their function and/or required musculoskeletal treatment from a health professional. Participants were also excluded from both groups if they were taking strong pain medications such as opioids. Non-steroidal anti-inflammatory drugs (NSAIDs) and paracetamol were not a reason for exclusion, but participants were asked not to take these drugs on the day of the experiment. Potential participants were screened by telephone, and were invited to attend a baseline evaluation appointment if eligible.

### Experimental protocol

Participants performed the following tasks in random order: A) Bilateral anterior shoulder flexion (SF) reaching 90 degrees and holding for 5s; movement symmetry was ensured by asking participants to hold a wooden bar horizontally; B) Anterior trunk flexion (TF), reaching the patellae with the tip of the fingers and standing up in 5s. Participants were asked to repeat the task if any degree of knee flexion was observed by the experimenter; C) Anterior pelvic tilt (APT) and back to neutral spine position in 5s. The task was performed in a sitting position, and participants were instructed to anteriorly tilt their pelvis as much as possible while avoiding movements of the thoracic spine. An examiner placed a hand behind the participant’s back to warn them when to stop the movement when returning to neutral position; D) Sit-to-stand (SS1 and SS2, see below) with arms hanging freely alongside their trunk; E) Walking (G1 and G2, see below) in a straight line for 10 meters at a self-selected pace. Each task was repeated 5 times. The tasks were standardized by using the same chair without armrests for all participants and by providing identical instructions. In addition, prior to testing, a researcher demonstrated the requested movement and asked participants to try it at least once. Two physiotherapists (F.S. and A.O.) inspected the execution of movements, ensuring that participants performed the task as instructed. These tasks were chosen to be representative of different daily-living activities that required the activation of the thoraco-lumbar muscles to different extents and in different postures.

### Experimental setup

**Fig. 1:**
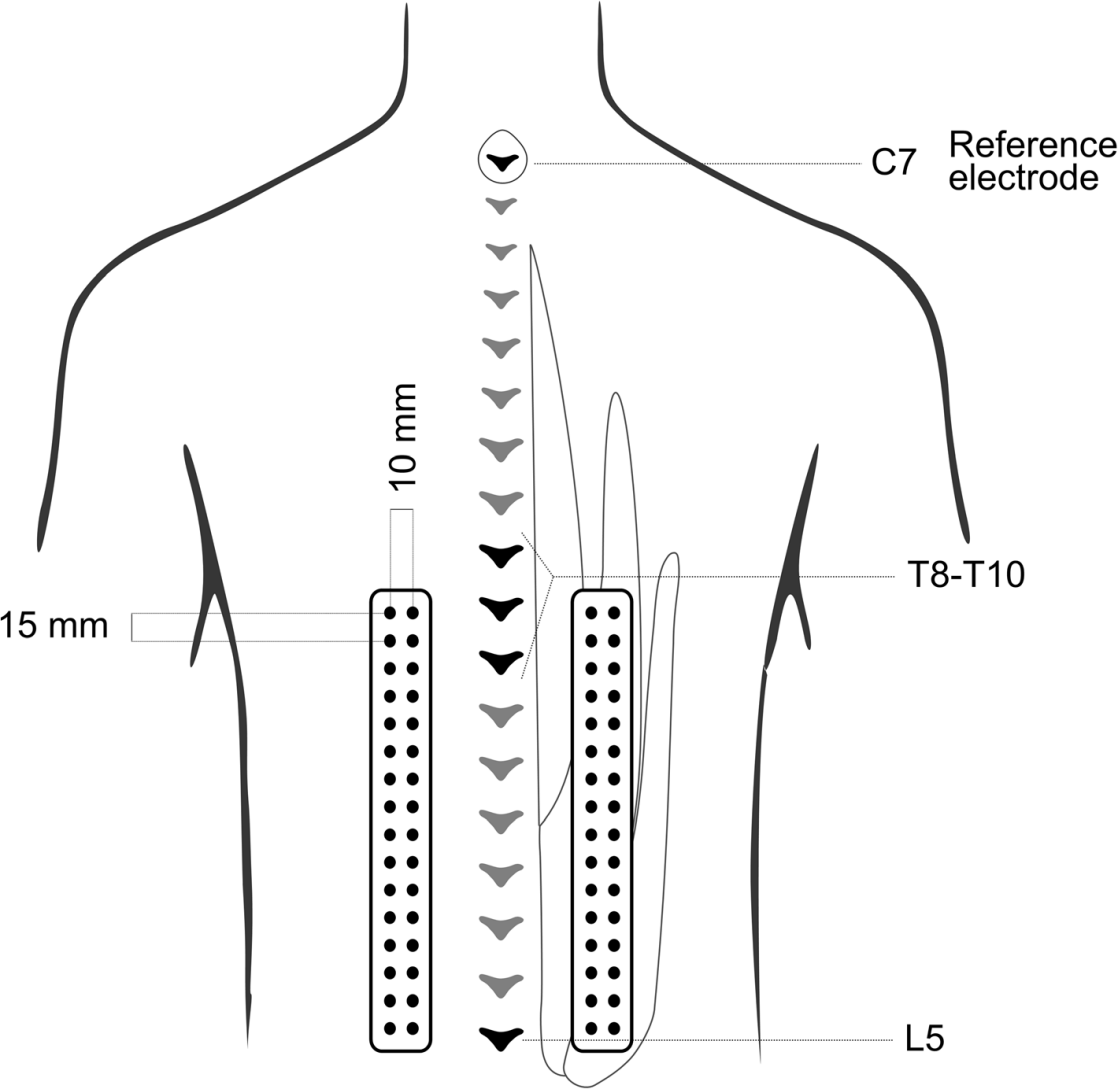
Experimental setup. The picture depicts the positioning of the HDEMG grids over the thoraco-lumbar extensor muscles in relation to the spine and the muscle mass. L5 location was identified through palpation and marked on the skin. Two grids of 32 surface electrodes were positioned one of each side of the spine. Each grid was placed 2–3,5 cm lateral to the spinous processes

Thoraco-lumbar muscle activation was assessed using HDsEMG. Surface HDsEMG signals were recorded using two semi-disposable grids of 16 × 2 silver circular electrodes each (1 mm diameter, center-to-center distance: 15 mm along and 10 mm transverse to the spine). Each grid was centrally located on the thoraco-lumbar muscle region identified through palpation during a lumbar extension movement. The electrode grids were placed on the bulk of the erector spinae muscle group identified by palpation, approximately 2-3.5 cm lateral to the vertebral spinous processes, and covered the thoraco-lumbar region from L5 (identified via palpation) to T8-T10 depending on the participant’s trunk length. A reference electrode was placed over the 7th cervical vertebra after skin preparation (Fig. 1). The electrode grids were secured to the skin using double-sided adhesive foam pad (Futura S.r.l. – Melzo, Italy) and holes in correspondence of the electrodes were filled with an electro-conductive paste to ensure electrode-skin contact. Before positioning the grids, the skin was shaved and treated with abrasive paste (NuPrep, Skin Prep Gel). HDsEMG signals were recorded in monopolar configuration using a recently developed acquisition system [24]. The system is composed of two miniaturized modules, each sampling 32 monopolar EMG signals. After amplification (183 V/V) and bandpass filtering (10 Hz – 500), EMG signals were sampled at 2048 Hz, digitized with 16 bit resolution and transmitted wirelessly to a laptop.

Twin-axis SG150 electrogoniometers (Biometrics Ltd, Newport, UK) were secured to the lumbar spine, to the right knee and to the right shoulder for measuring motion on the sagittal plane. For the lumbar spine, the proximal base of the goniometer was secured on the skin overlying the L1-L4 spinous processes, and the distal base was fixed over the sacrum. On the knee, the goniometer was mounted on the lateral side of the leg while the participant was standing. For the shoulder, the proximal base was secured to the skin on the humerus, and the distal base was fixed over the pectoralis major parallel to the sternum. The electrogoniometers were connected to a wireless system for the acquisition of biomechanical signals (DueBio, OT Bioelettronica, Turin, Italy) and collected using the same acquisition system used for the HDsEMG.

### Patient-reported outcome measures

Prior to testing, participants self-reported information on anthropometrics, medical history, and duration of pain. Participants from both groups completed patient-reported outcome measures (PROMs) included in the core outcome measurement set for LBP [25] and to evaluate key psychosocial traits known to be prognostic factors and/or mediators in the link between pain and disability [26, 27]. The following PROMs were administered:

- the Oswestry Disability Index (ODI) version 2.1a to assess LBP-related physical functioning;
- an 11-point Numeric Rating Scale (NRS) to evaluate average pain intensity over the last week;
- the 10-item Patient-Reported Outcome Measurement Information System Global Health short form (PROMIS-GH) to measure Health-Related Quality of Life (HRQoL);
- the Modified Somatic Perception Questionnaire (MSPQ) to evaluate somatization;
- the Hospital Anxiety and Depression Scale (HADS) for anxiety and depression;
- the Pain Catastrophizing Scale (PSC) for pain catastrophizing;
- the Pain Self-Efficacy Questionnaire (PSEQ) for pain self-efficacy;
- the Fear Avoidance Beliefs Questionnaire (FABQ) to evaluate fear-avoidance beliefs.

### Data analysis

**Fig. 2:**
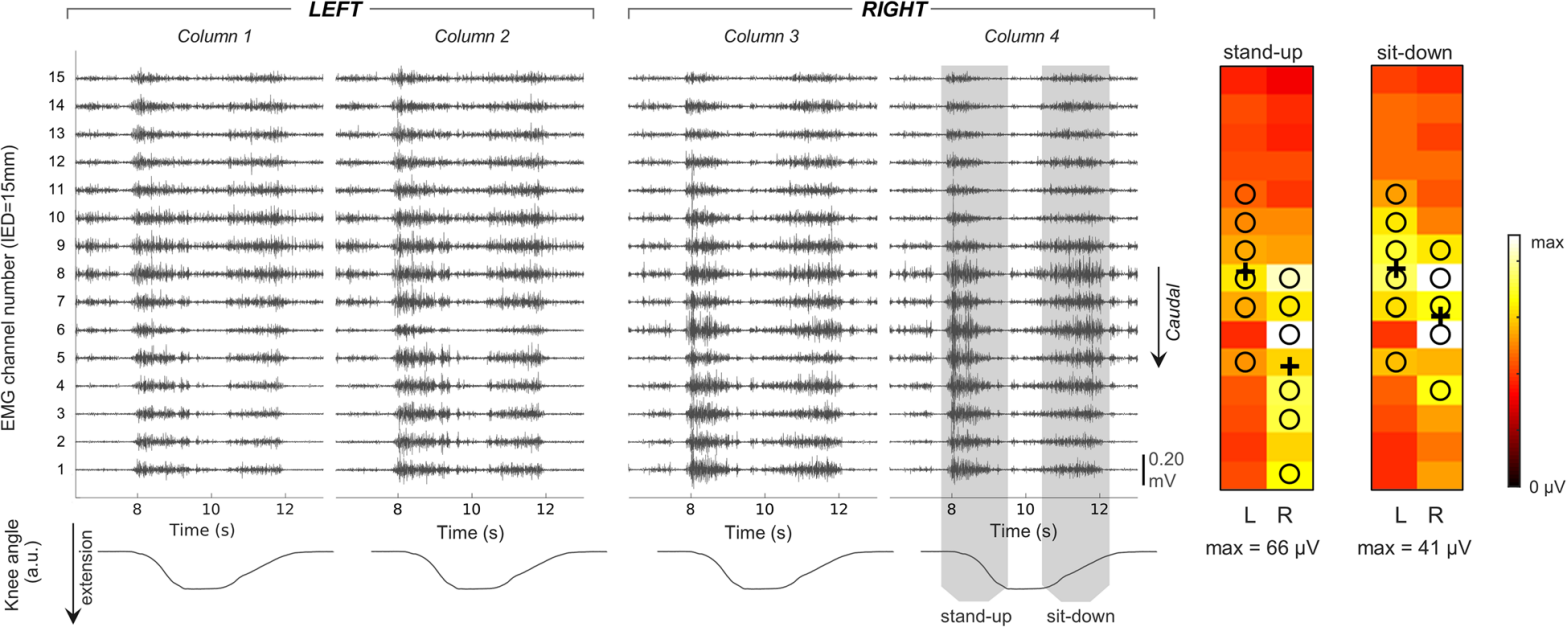
*On the left*: activation of the thoraco-lumbar extensor muscles for a single repetition of sit to stand task. Raw surface, single-differential EMGs (black traces) are depicted in the top row for each of the 60 channels separately. The knee kinematics, used to identify the identify the stand-up and sit-down phase of the movement, is depicted in the bottom row. *On the right*: an example of activity distribution during sit to stand task (Root Mean Square values averaged over five task repetitions). The centroid of channels is marked with a black cross; black circles indicate channels with amplitude > 70% of the maximum. The scale of each colormap is indicated in µV

Data were analyzed using a custom MATLAB script (The Mathworks Inc., USA). Monopolar EMG signals were offline digitally filtered between 20–400 Hz (Butterworth, 4th order) and differentiated along the cranio-caudal direction, providing 30 single-differential channels each side. Signal quality was determined by visual inspection, and channels showing large movement artefacts, power line interference or baseline noise were excluded and replaced by the linear interpolation of the neighboring channels. An example of raw surface EMG signals is shown in (Fig. 2).

**Fig. 3:**
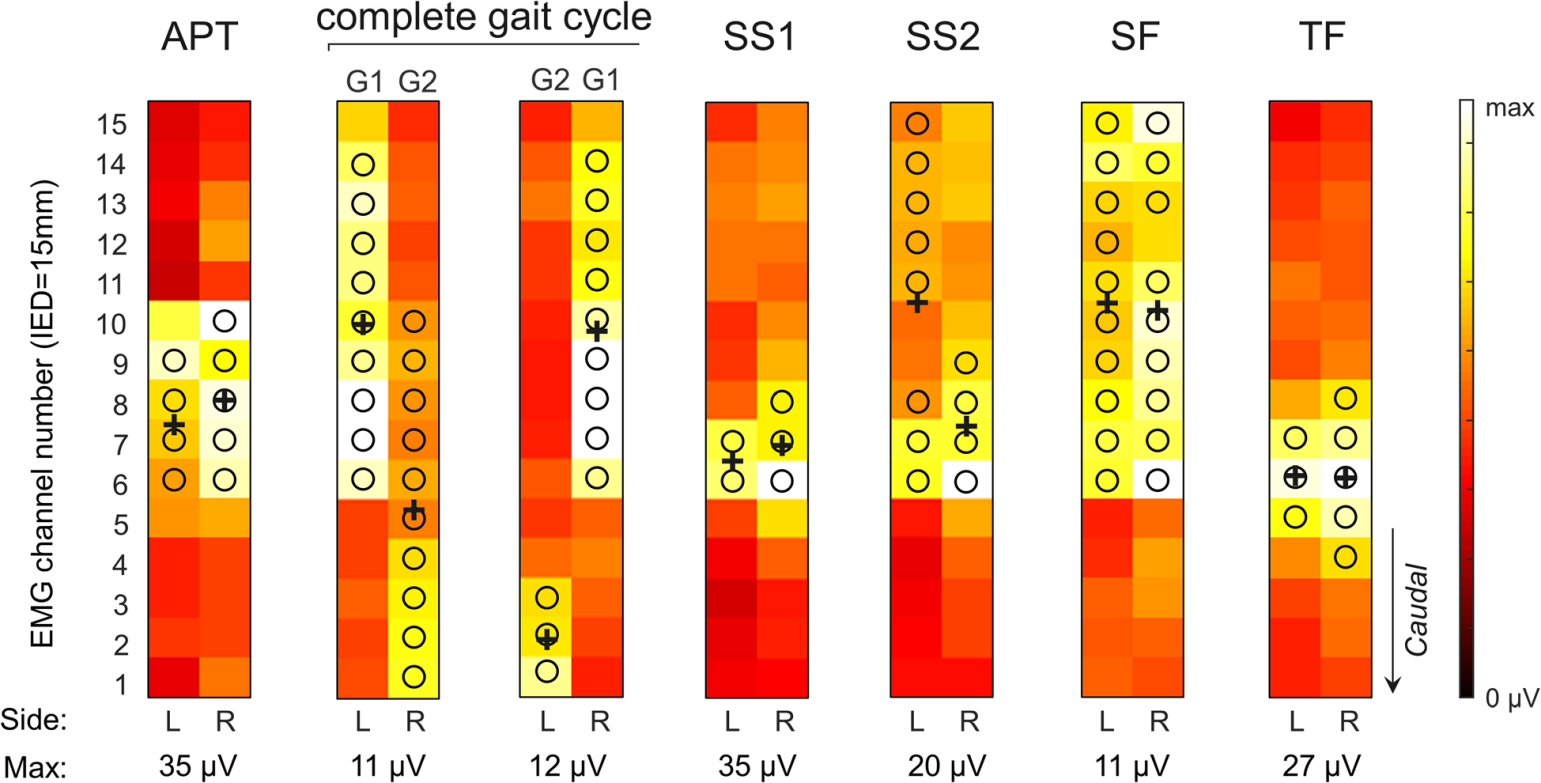
EMG amplitude distribution during different functional tasks (APT, Anterior Pelvic Tilt; G1, loading response phase of gait; G2, pre-swing phase of gait; SS1, stand up; SS2, sit down; SF, shoulder flexion; TF, trunk flexion) in a healthy participant

Individual repetitions within each task were identified through segmentation of joint angles. Cycles were time-normalized between 0 (start of each repetition of a task) and 100% (end of each repetition) and averaged across the 5 repetitions. Gait cycles were identified from the peak of knee flexion angle. EMG envelopes were calculated by low-pass filtering (Butterworth, 4th order, 10 Hz) the full-wave rectified, single-differential EMGs. Based on the kinematics, EMG envelopes were also time-normalized between 0-100% and averaged across repetitions, separately for each channel. A large between-subject variability in the relation between joint kinematics and lumbar muscle activation was observed, especially in the shoulder flexion and anterior pelvic tilt tasks. Moreover, during pelvic tilt we often observed the angle data did not reflect the actual movement amplitude, likely because inter-individual anthropometric differences resulted in different locations for the proximal base of the electrogoniometer at the spine. We therefore defined the epoch for analysis based on individual profiles of muscle activation. For each participant and task, the overall activation profile in time of the lumbar extensor muscles was obtained by averaging EMG envelopes across channels and sides. A single activity peak was clearly observed in the Trunk Flexion, Shoulder Flexion and Anterior pelvic Tilt tasks, whereas two activity peaks were identified for Gait and Sit-To-Stand; these tasks are named stand-up (SS1) and sit-down (SS2) for Sit-To-Stand, and loading response (G1) and pre-swing (G2) for Gait. Each EMG envelope was averaged over a window spanning 10% of the duration of the task, centered around the peak of the overall activation profile, resulting in a 15x4 matrix of EMG amplitude values. For each side, amplitude estimates were averaged across the two columns, resulting in 15 amplitude estimates along the cranio-caudal direction per side. Each EMG amplitude distribution was characterized in terms of amplitude, size and location [28], separately for the left and right sides. To characterize and compare the EMG amplitude distribution between and within participants, the channels with amplitude values higher than 70% of the maximum were segmented in a single cluster characterized in terms of size (number of channels), location (centroid in the cranio-caudal location) and amplitude (average amplitude) (Fig. 3). EMG amplitude values were compared between groups both as absolute values and normalized to the following submaximal tasks: SF (most standardized task), SS1 (task that resulted in the highest EMG amplitude values) and TF (task requiring the most selective activation of the thoraco-lumbar extensors). The centroid was calculated in a similar way as in previous studies [29], but only for the segmented channels to have centroid values closer to the location of channels where large activation could be observed [28]. The cranio-caudal location of the centroid has been previously shown to be highly reliable between days when calculated from HDsEMG recordings from the lumbar extensors [30]. Given there was no expectation as per which trunk side should present greater EMG amplitudes distributed over a greater region, side differences were assessed as the absolute difference between values from the right and left sides for each of the three EMG indices. The resulting difference was then normalized with respect to the average of the two values for amplitude and size comparisons. Side comparisons and asymmetries were calculated for EMG estimates obtained at the same time instants of the kinematic cycle for symmetrical tasks (anterior pelvic tilt, trunk flexion, sit-to-stand and shoulder flexion). As gait required an asymmetrical activation of the thoraco-lumbar extensors, side comparisons and asymmetries were calculated for EMG estimates obtained during the same phase of the gait cycle (e.g.: activation of the left thoraco-lumbar extensors during stance phase on the left leg compared to activation of the right thoraco-lumbar extensors during stance phase on the right leg; Fig. 3).

### Statistical analysis

Statistical analysis was performed using SPSS v. 26 (IBM Inc., Armonk, NY, USA). An a-priori sample size calculation was performed using GPower [31] based on the use of independent Student T-test. Assuming a large effect size (Cohen’s d 0.8) based on previous publications [15, 18], a power of 0.80, and a statistical significance level of 0.05, 21 participants per group were required. Descriptive characteristics were summarized as mean and standard deviation or median and inter-range quartiles, depending on data distribution. Parametric statistics or non-parametric statistics were used depending on whether the data residuals were normally distributed (Shapiro-wilk test), and whether other test-specific assumptions were verified (Levene’s test, Box’s test). Logarithmic transformation of the data was applied if data did not display a normal distribution. When the assumption of sphericity was violated, Greenhouse-Geisser correction was applied. Three-way ANOVAs were used to determine the effect of task (7 levels, within-subject factor), side (2 levels, within-subject factor) and group (2 levels, between-subject factor) separately for activation intensity and location. Bonferroni correction was applied to post-hoc comparisons. Statistical analyses on the location of activation were also performed after normalizing the location values by each participant’s height to account for differences in the spinal levels included under the HDsEMG grid; as no differences were observed in the statistical significance of the results, only statistics for the non-normalized values are reported. Multiple independent Wilcoxon Mann-Whitney tests were applied to compare size, amplitude asymmetry, size asymmetry and location asymmetry between LBP and control, separately for each task. A Bonferroni correction was applied by multiplying the *p* value by the number of comparisons.

Pearson’s correlation coefficients were used to assess the relationship between patient-reported information and EMG descriptors. Correlations were only calculated in the LBP group for the EMG descriptors that differed between LBP and controls. Correlations were investigated with four PROMs, as two of them (i.e. ODI and NRS) are the strongest prognostic factors for poor long-term outcomes in LBP [26], and the other two (PCS and PSEQ) are the strongest prognostic factors among various psychosocial domains [32]; additionally, the (total) scores of these PROMs are sufficiently unidimensional to be used as continuous variables in correlation analyses [33–35]. Pearson’s correlation coefficient values ranging from 0 to 0.35, from 0.36 to 0.67 and from 0.68 to 1 were regarded as “weak”, “moderate” and “high”, respectively. The statistical significance was set at *p* value < 0.05.

## Results

Twenty-one participants per group were included. Demographic characteristics did not differ between the groups (all *p* > 0.05; Table 1). As expected, PROM scores for the clinical domains differed between individuals with and without LBP (Table 1). Across participant and tasks, few channels were replaced because of poor signal quality (median 0, range: 0–3). The muscle activation peaks were identified at comparable instants of the kinematic cycles for EMG envelopes in both groups: (25th – 75th percentiles; participants without LBP, G1: 24–29%; G2: 74–78%; APT: 25–68%; TF: 66–82%; SS1: 5–9%; SS2: 76–83%; SF: 16–32%; participants with LBP: G1: 22–26%; G2: 72–75%; APT: 30–56%; TF: 68–81%; SS1: 4–6%; SS2: 78–85%; SF: 14–23%).

**Table 1:** Characteristics of the LBP and control groups. Values are presented as mean (SD)

**Characteristic**	**Controls**	**LBP**	*p ***value**
Sex	11 female, 10 male	11 female, 10 male	
Mean age (years)	39.33±13.25	43.57±12.55	*p*=0.294
BMI	22.77±2.10	23.18±3.20	*p*=0.629
ODI (%)	0.95±2.33	15.57±7.15	*p*<0.001
NRS	0	4.43±1.54	/
Pain duration (months)	0	60.29±95.87	/
TSK			
TSK/1	6.9±1.4	9.8±3.4	*p*=0.001
TSK/2	10.1±3.4	14.4±4.2	*p*=0.001
FABQ			
Work	2.19±5.67	15.81±10.62	*p*<0.001
Physical activity	1.95±3.68	10.43±6.37	*p*<0.001
PSEQ	59.19±2.14	51.90±7.91	/
PCS	4.57±6.85	11.86±6.89	/
HADS			
Anxiety	1.86±1.62	5.33±2.89	*p*<0.001
Depression	0.81±1.03	2.67±1.77	*p*<0.001
MSPQ	1.57±1.94	6.10±4.70	*p*<0.001
PROMIS Global Health			
Physical	59.71±6.26	45.00±6.65	*p*<0.001
Mental	55.65±7.64	51.57±6.62	*p*=0.072

*BMI*, Body Mass Index, *ODI *Oswestry Disability Index, *NRS *Numeric Rating Scale, *TSK *Tampa Scale of Kinesiophobia, *FABQ *Fear Avoidance Belief Questionnaire, *PSEQ *Pain Self-Efficacy Questionnaire, *PCS *Pain Catastrophizing Scale, *HADS *Hospital Anxiety and Depression Scale, *MSPQ *Modified Somatic Perceptions Questionnaire, PROMIS Global Physical and Mental Health

### Amplitude of the HDsEMG cluster

**Fig. 4:**
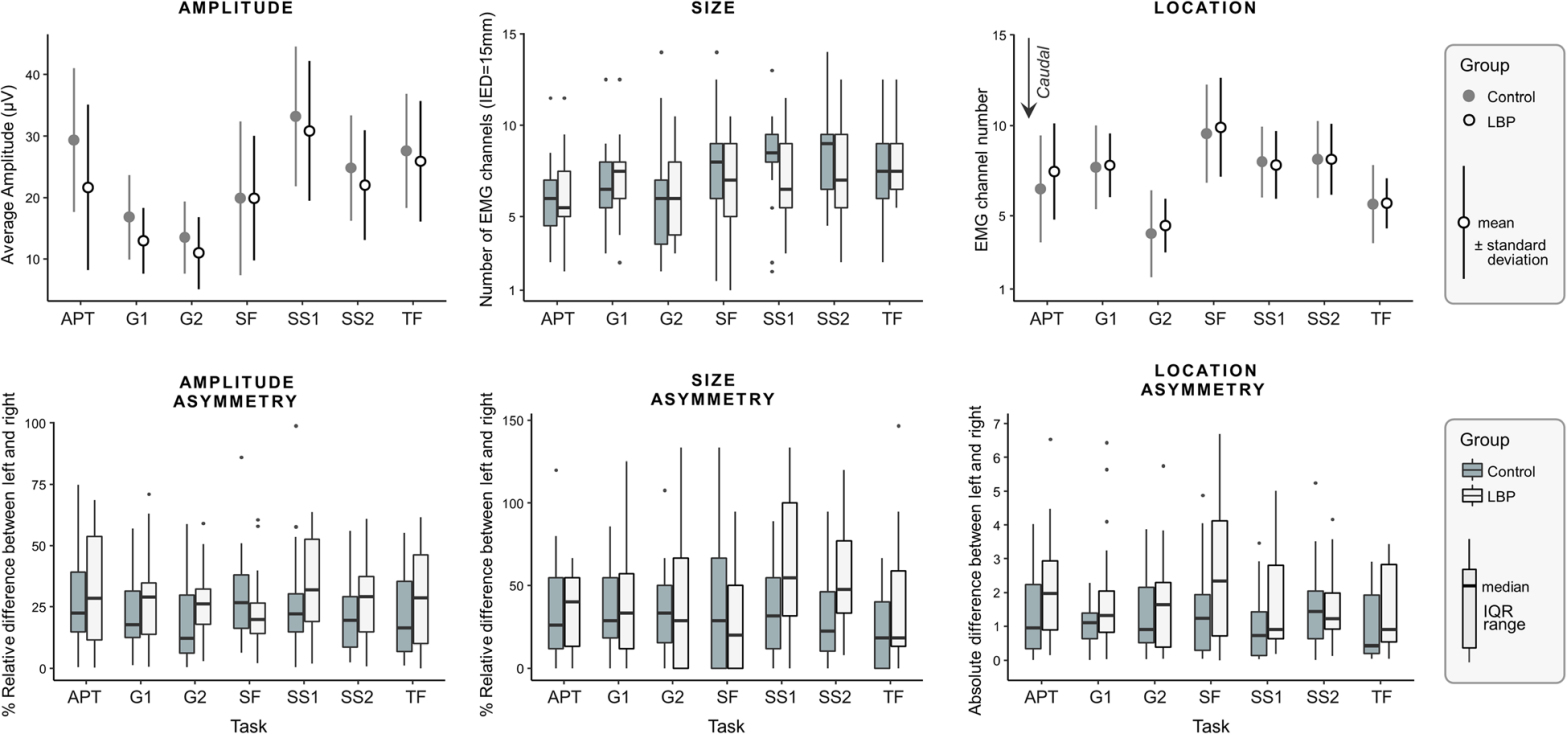
Descriptors of EMG amplitude distribution and side asymmetry indices. *Top row*: mean values and standard deviation for each task (APT, anterior pelvic tilt; G1, loading response phase of gait; G2, pre-swing phase of gait; SS1, stand up; SS2, sit down; SF, shoulder flexion; TF, trunk flexion) and group (LBP and control), left and right side averaged. *Bottom row*: side asymmetry indices by task and group

The 3-way ANOVA identified a significant interaction between ‘task’ and ‘group’ (*F*(6,240) = 2.976, *p* = 0.008) and an interaction between ‘task’ and ‘side’ (*F*(4.302,172.074) = 2.506, *p* = 0.04). Post-hoc comparisons identified a significant between-group difference for APT and G1; specifically, EMG amplitude was 26% lower (*p* = 0.018; mean difference: 0.42, 95% confidence interval: [0.07, 0.76]) in people with LBP (21.7 ± 14.5 µV) compared to people without LBP (29.4 ± 12.9 µV) during the anterior pelvic tilt task, and 20% lower (*p* = 0.035; 0.26 [0.20, 0.50]) during loading response phase (LBP: 12.4 ± 6.6 µV; without LBP: 15.6 ± 7.2 µV; Fig. 4). Statistical significance was similar when the absolute EMG values were normalized to SF (APT: *p* = 0.007, G1: *p* = 0.02), SS1 (APT: *p* = 0.016, G1: *p* = 0.069, trend) or TF (APT: *p* = 0.013, G1: *p* = 0.044). No other between-group comparisons were significant after Bonferroni correction (*p* > 0.107). Post-hoc testing revealed that the interaction effect between ‘task’ and ‘side’ was mainly driven by a larger activation of the left compared to right thoraco-lumbar extensors during SS1 (*p* = 0.025; 0.13 [0.02, 0.24]). Significant differences in EMG amplitude between tasks were generally similar between the left and right side; for this reason, the pairwise posthoc comparisons of the main effect ‘task’ are reported in Table 2. Amplitude asymmetry did not differ between individuals with or without LBP for any of the tasks considered (Bonferroni-corrected multiple *p* > 0.406).

**Table 2:** Mean difference and 95% confidence interval between functional tasks for amplitude (bottom-left) and location (top-right)

POS	G1	APT	TF	SS1	SF	G2	SS2
AMP							
G1		0.77 [-0.27, 1.81]	**2.08 [1.52, 2.63]**	-0.16 [-0.96, 0.64]	**-1.98 [-3.27, -0.69]**	**3.51 [2.63, 4.39]**	-0.39 [-1.19, 0.41]
APT	**-0.47 [-0.67, -0.26]**		**1.30 [0.23, 2.38]**	-0.93 [-2.12, 2.65]	**-2.75 [-4.44, -1.05]**	**2.74 [1.64, 3.83]**	-1.16 [-2.40, 0.75]
TF	**-0.61 [-0.75, -0.46]**	-0.14 [-0.35, 0.07]		**-2.23 [1.49, 2.98]**	**-4.05 [-5.27, -2.83]**	**1.43 [0.71, 2.16]**	**-2.47 [-3.24, -1.69]**
SS1	**-0.78 [-0.94, -0.63]**	**-0.31 [-0.53, -0.09]**	**-0.18 [0.32, 0.03]**		**-1.82 [-3.12, -0.52]**	**3.67 [2.69, 4.64]**	-0.23 [-0.69, 0.23]
SF	**-0.24 [-0.44, -0.04]**	0.23 [-0.03, 0.48]	**0.37 [0.16, 0.57]**	**0.54 [0.32, 0.76]**		**5.48 [4.19, 6.77]**	**1.58 [0.39, 2.78]**
G2	**0.22 [0.06, 0.37]**	**0.69 [0.42, 0.95]**	**0.82 [0.63, 1.01]**	**0.99 [0.78, 1.21]**	**0.46 [0.23, 0.69]**		**-3.89 [-4.91, -2.89]**
SS2	**-0.47 [-0.62, -0.31]**	0.01 [-0.22, 0.22]	0.14 [-0.01, 0.29]	**0.32 [0.20, 0.43]**	**-0.26 [-0.44, -0.01]**	**-0.68 [-0.90, -0.47]**	

Bold characters identify statistically significant comparisons. Amplitude values are reported after logarithmic transformation

*APT *Anterior Pelvic Tilt, *G1 *Gait stance phase, *G2 *Gait swing phase, *SS1 *Stand-up, *SS2 *Sit-down, *SF *Shoulder flexion, *TF *Trunk flexion

### Location of the HDsEMG cluster

The 3-way ANOVA revealed that the location of the segmented cluster was not different between groups (main effect of group: *F*(1,40) = 0.223, *p* = 0.639; interactions: *F* < 0.673, *p* > 0.672). A significant interaction between ‘location’ and ‘side’ was identified (*F*(4.058,162.338) = 2.620, *p* = 0.036). Post-hoc comparisons identified a more cranial amplitude distribution on the right side during SS1 (*p* = 0.009, -0.76 [-1.3, -0.2]). Besides this, the location of the cluster between tasks were generally similar between the left and right side; for this reason, the pairwise posthoc comparisons of the main effect ‘task’ are reported in Table 2. Asymmetries in the cranio-caudal location of clusters did not differ significantly between individuals with or without LBP for any of the tasks (Bonferroni-corrected multiple *p* > 0.280).

### Size of the HDsEMG cluster

Multiple independent Wilcoxon tests did not identify between-group differences in the size of the cluster for any tasks or side (*p* > 0.138). Size asymmetry did not differ significantly between individuals with or without LBP for any of the tasks (Bonferroni-corrected multiple *p* > 0.112).

### Association between EMG amplitude distribution and clinically relevant domains

**Fig. 5:**
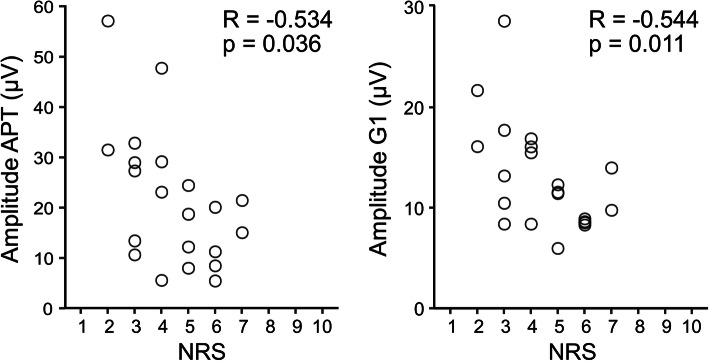
Correlations between pain intensity (NRS, Numeric Rating Scale) and EMG amplitude during anterior pelvic tilt (left) during G1 task (loading response phase of gait, right). Each circle represents a different participant

Pearson’s correlation coefficient indicated a moderate inverse association between pain intensity and EMG amplitude during loading response phase (*R*=-0.54, *p* = 0.011) and anterior pelvic tilt (*R*=-0.53, *p* = 0.036), indicating that people with higher LBP display lower thoraco-lumbar extensors activation during these tasks. No other significant associations were identified between EMG amplitude and ODI, PCS and PSEQ (all *R* < 0.27, all *p* > 0.225). (Fig. 5).

## Discussion

This study showed that, compared to asymptomatic participants, individuals with chronic LBP demonstrated reduced amplitude of thoraco-lumbar muscle activation during anterior pelvic tilt and during loading response phase of gait tasks. These neuromuscular alterations were especially evident in participants who reported higher pain intensity. No associations were found between EMG amplitude and the other PROMs.

Differences in the cranio-caudal distribution of EMG amplitude can be due to differences in activation within the lumbar extensors [12]. The sensitivity of HDsEMG to changes in EMG amplitude distribution in thoraco-lumbar muscles is substantiated by differences in the cranio-caudal localization of EMG activity observed during different motor tasks. Neurophysiologically, our findings suggest that cranio-caudal regions within the thoraco-lumbar extensors are preferentially recruited in different tasks. This heterogeneous activation may reflect the activation of different muscles (superficial multifidus caudally and longissimus more cranially, according to their anatomy [36, 37]) as well as different regions within the longissimus due to its localized motor unit territories [38]. Building on evidence from previous studies showing regional activation during simple tasks such as trunk rotation [38] and postural perturbations [39–41], this study shows that lumbar muscles are activated regionally during functional tasks. Our results suggest that multiple electrodes may be needed to estimate the activation of the thoraco-lumbar extensors, especially when comparing EMG activation between tasks. During anterior pelvic tilt and loading response phase of gait tasks, the thoraco-lumbar muscle activity was found to be significantly higher in the control group than in the LBP group. While recent systematic reviews [42, 43] suggest an increase of erector spinae activity in individuals with LBP, most of the studies included in these reviews assessed walking on a treadmill. As there are known differences in trunk and pelvis kinematics between walking overground and on a treadmill [44], the results of this study cannot be directly compared to those reported in the aforementioned systematic reviews. In the current study, we observed a general decrease of thoraco-lumbar EMG amplitude during gait, which may reflect altered motor control to stabilize the trunk during weight transfer. Regarding the anterior pelvic tilt task, reduced EMG amplitude may indicate that LBP participants had poor ability to dissociate movements at different spine regions (poor intersegmental coordination), as shown by impaired performance of the thoraco-lumbar dissociation test [45, 46]. Our findings suggest that individuals with LBP asked to perform a pelvic anteversion task activate their thoraco-lumbar muscles less than individuals without LBP when allowed to do so. Whether these adaptations are precursors or results of LBP has to be determined in future studies.

While previous studies have shown altered regional activation of the lumbar erector spinae [13–16, 18] in people with LBP performing tasks that require high activation levels, our study identified no differences in regional activation between groups during low-effort functional tasks. This is consistent with the observation of similar spatial variability in EMG amplitude between individuals with and without LBP during a sustained sitting task [17]. Taken together, these findings suggest that altered regional activation in LBP may be task-specific, preferentially present during higher effort or fatiguing tasks, potentially when spinal stability is challenged. Similar to a previous study [19], we observed asymmetric EMG amplitude distributions over the thoraco-lumbar extensors in healthy participants, even when performing symmetrical tasks. Indices of asymmetry did not differ between individuals with and without chronic LBP. While earlier studies using bipolar EMG reported that individuals with LBP demonstrate more side-to-side differences in EMG amplitude values when performing maximal contractions of the lumbar extensors [47], evidence from submaximal contractions is conflicting [48–51]. Our findings, strengthened by the fact that muscle activation was collected from several locations over the thoraco-lumbar extensors, support the notion that asymmetrical muscle activation is less prevalent in individuals with LBP performing submaximal tasks.

The moderate inverse associations between pain intensity and EMG amplitude during anterior pelvic tilt and loading response phase of gait suggest that the extent of motor adaptation to pain may be in part related to pain intensity, with individuals with LBP reporting more intense pain showing lower activation of their thoraco-lumbar extensors. Another study reported that individuals with higher pain intensity demonstrate lower ability to redistribute activation within their lumbar extensors [14]. Nevertheless, our findings should be evaluated in light of the exploratory nature of our study in studying associations between EMG amplitude and clinical domains. Future studies evaluating EMG amplitude in patients with chronic LBP should also assess other clinical features, to further explore the (lack of) associations observed in the current study.

This study has some limitations. The sample size was calculated to detect differences between groups with a large effect size, therefore the lack of statistical significance for some comparisons may be due to insufficient power. Despite chronic LBP symptoms, our population reported low levels of disability. Our findings may therefore not be generalizable for people with LBP and higher disability. While there is evidence that similar estimates of centroid location of regional thoraco-lumbar extensors activation can be obtained in different days, it is currently unknown whether estimates of intensity of activation and size of the cluster are reliable between days. Our results have been interpreted considering the amplitude of surface EMG to be directly related to the actual degree of lumbar muscle excitation in the two groups. However, this assumption may hold only in very controlled situations [52]. In an attempt to reduce the effect of factors unrelated to muscle excitation, EMG amplitude values during submaximal tasks are usually normalized to those recorded during maximal contractions. However, individuals with LBP may be unable to fully activate their lumbar muscles due to pain during maximal voluntary contraction tasks; furthermore, repeated maximal voluntary contractions may exacerbate LBP symptoms. Therefore, we decided to compare non-normalized EMG amplitude values in line with other studies [15, 18]. In addition, statistical significance was similar when normalizing the EMG amplitude collected during APT and G1 to other submaximal tasks. Our interpretation that the observed differences in EMG amplitude between groups at least partly reflect differences in muscle excitation is supported by the fact that differences were observed in some (G1 and APT; Fig. 4), but not all tasks. While we understand future studies are necessary to systematically account for anatomical differences between groups, we believe that between-group differences in muscle excitation are the most likely explanation for our results.

## Conclusions

Our findings suggest that side differences in regional activation of the thoraco-lumbar extensors are comparable in individuals with and without LBP. Reduced thoraco-lumbar muscle activation during anterior pelvic tilt and loading response phase of gait was observed in people with LBP, especially those who reported higher pain intensity. While differences in regional distribution of EMG activity were clearly observed between tasks, these spatial distributions were similar between people with and without LBP, indicating that participants recruited similar regions of the thoracolumbar extensors regardless of the presence of LBP. This knowledge provides new insights into impaired motor control of spinal muscles in individuals with chronic LBP, which may be useful in the development of new assessments and interventions focusing on motor control in patients with chronic LBP.

## Data Availability

The datasets used and/or analysed during the current study are not publicly available because publication of the dataset was not included in the consent form signed by the participants of the study, but are available from the corresponding author on reasonable request.

## Abbreviations

APT: Anterior Pelvic Tilt
EMG: Electromyography
FABQ: Fear Avoidance Beliefs Questionnaire
G1: Loading response phase of Gait
G2: Pre-swing phase of Gait
HADS: Hospital Anxiety and Depression Scale
HDsEMG: High Density Electromyography
HRQoL: Health-Related Quality of Life
LBP: Low Back Pain
MSPQ: Modified Somatic Perception Questionnaire
NRS: Numeric Rating Scale
ODI: Oswestry Disability Index
PCS: Pain Catastrophizing Scale
PSEQ: Pain Self-Efficacy Questionnaire
PROMIS-GH: Patient-Reported Outcome Measurement Information System Global Health short form
SF: Shoulder Flexion
SS1: Sit-to-Stand, stand up
SS2: Sit-to-Stand, sit down
TF: Trunk Flexion
